# Direct Evidence of Lack of Colocalisation of Fluorescently Labelled Gold Labels Used in Correlative Light Electron Microscopy

**DOI:** 10.1038/srep44666

**Published:** 2017-03-20

**Authors:** Benjamin T. Miles, Alexander B. Greenwood, David Benito-Alifonso, Hugh Tanner, M. Carmen Galan, Paul Verkade, Henkjan Gersen

**Affiliations:** 1 Nanophotonics and Nanophysics Group, H.H. Wills Physics Laboratory, University of Bristol, Bristol, BS8 1TL, UK; 2 School of Chemistry, University of Bristol, Cantock’s Close, Bristol, BS8 1TS, UK; 3 Bristol Centre for Functional Nanomaterials, H.H. Wills Physics Laboratory, University of Bristol, Bristol, BS8 1TL, UK; 4 Wolfson Bioimaging Facility, University of Bristol, Bristol, BS8 1TD, UK; 5 School of Biochemistry, University of Bristol, Bristol, BS8 1TD, UK

## Abstract

Fluorescently labelled nanoparticles are routinely used in Correlative Light Electron Microscopy (CLEM) to combine the capabilities of two separate microscope platforms: fluorescent light microscopy (LM) and electron microscopy (EM). The inherent assumption is that the fluorescent label observed under LM colocalises well with the electron dense nanoparticle observed in EM. Herein we show, by combining single molecule fluorescent imaging with optical detection of the scattering from single gold nanoparticles, that for a commercially produced sample of 10 nm gold nanoparticles tagged to Alexa-633 there is in fact no colocalisation between the fluorescent signatures of Alexa-633 and the scattering associated with the gold nanoparticle. This shows that the attached gold nanoparticle quenches the fluorescent signal by ~95%, or less likely that the complex has dissociated. In either scenario, the observed fluorescent signal in fact arises from a large population of untagged fluorophores; rendering these labels potentially ineffective and misleading to the field.

Combining the advantages of light and electron microscopy, Correlative Light Electron Microscopy (CLEM) offers a unique tool set for the tracking and localisation of biological labels to gain insight into biological systems^1^. Light microscopy provides the ability for wide field or confocal imaging typically of live cells and localisation of fluorescent labels *in vitro* which may be extended into super resolution techniques^2^, while electron microscopy enables higher resolution imaging of the same area or event and further localisation through the use of electron dense labels within the cellular ultrastructure^3^,^4^. Commonly selected labels for CLEM combine an electron dense nanoparticle, such as gold, and a fluorophore^1^,^5^; although there is evidence that direct attachment of fluorophores to gold nanoparticles may result in the quenching of the fluorescent signal as a result of resonant energy transfer^6^,^7^,^8^,^9^. The potential for quenching behaviour presents a scenario where colocalising these biological labels under CLEM may be prone to misinterpretation of the results in the presence of much smaller and hence more pervasive unbound fluorescent molecules.

Assessment of this quenching behaviour has to date largely been conducted by fluorometric analysis of the bulk probe solution^10^, however it is important to conduct this analysis at the single CLEM label level to remove any ambiguity in the analysis that may arise from fluorescent contributions from unbound fluorophores. To directly assess the fluorescent signal colocalised with individual CLEM probes, we use a recently introduced technique, Interferometric Cross-Polarization Microscopy (ICPM), which has shown detection of the weakly scattered signal from individual metallic nanoparticles down to 5 nm at low optical powers (<1 μW)^11^. This makes ICPM compatible with simultaneous fluorescent collection with single molecule sensitivity^12^. Using this approach, we have already shown detection of the weak scattering signature of 10 nm nanodiamond colocalised with the fluorescent signature from embedded *NV*^−^ centers; optical active lattice defects^12^. In this paper, we investigate one of a variety of available CLEM labels that use a fluorescent moiety that has been tagged with a small gold particle, in our case, streptavidin labelled with Alexa Fluor 633 (Life Technologies/Thermo Fisher) and coupled to 10 nm gold by Aurion (Aurion, Wageningen, The Netherlands). We show through combining single molecule fluorescent detection with optical detection of the scattering from single gold nanoparticles that there is in fact a complete lack of colocalisation of the signals.

## Results and Discussion

**Figure 1:**
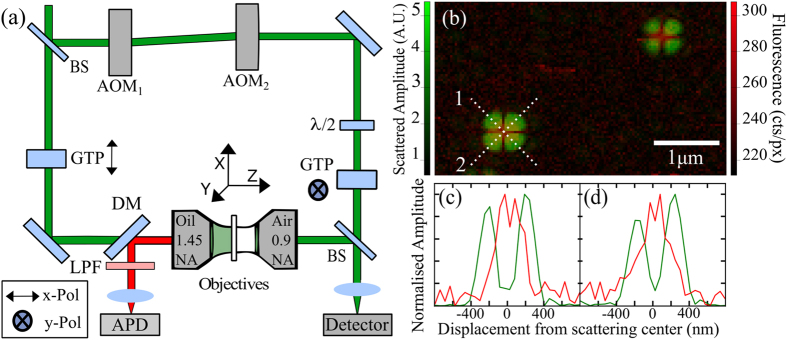
(**a**) Schematic diagram of interferometric cross-polarization microscope (ICPM) that combines the ability to detect scattering of single nanoparticles and single molecule fluorescence, further details in main text. (**b**) Overlayed false colour images of scattering and fluorescent signal (green and red respectively) produced by 10 nm fluorescent nanodiamond demonstrating the excellent overlap we achieve. The applied optical power for *λ* = 532 nm is 37.0 μW. Images are captured at a pixel (px) resolution of 36 nm/px × 39 nm/px over an imaging area of 4.5 μm × 2.9 μm at 124 pixels × 74 pixels collected at a scan rate of 0.24 s/line. (**c**,**d**) line scans 1 and 2 for one of the fluorescent nanodiamond particles in (**b**).

Optical detection of the scattering signal from small nanoparticles is performed *via* ICPM which operates in the crossed-polarized regime using interferometric enhancement of light scattered between two objectives to detect even weakly scattering signatures^13^. The technique is point-scanning and confocal-like in nature^14^ and arranged as schematically depicted in Fig. 1(a). A coherent light source is incident on a 50:50 beam splitter (BS) to generate a signal and reference branch, which are frequency offset and orthogonally polarized through the use of acousto-optic modulators (AOM), waveplates and Glan-Thompson Polarizers (GTP). Nano-objects in the focal volume scatter the polarized field generated by strong focusing of linearly polarized light^15^,^16^ where only scattered light polarized orthogonally to the incident illumination is interferometrically enhanced *via* the like-polarized reference branch^17^. Stokes-shifted fluorescent light collected by the illumination objective is reflected by a dichroic mirror (DM) and after passing through a longpass filter (LPF) collected on an avalanche photodiode (APD) (Perkin Elmer SPCM-AQRH-14) with a dark count rate of less than 100 counts per second (cts/s).

In order to optimise the detection sensitivity to the scattered signature of 10 nm gold nanoparticles, the sample is illuminated at 532 nm; close to the plasmon resonance where the scattering cross-section is maximised. To collect the fluorescent image, the illumination wavelength used is 632 nm, and Stokes-shifted fluorescent light is collected by the illumination objective and incident on the APD after passing through a 640 nm longpass filter. To prepare a sample of CLEM labels containing 10 nm gold nanoparticles bound with Alexa Fluor 633 (herein referred to as 10 nm NP-A-633) a 1.5 H (Marienfield) glass coverslip is surface-charged *via* a 10 minute acid wash in sulfuric-nitric acid at a ratio of 1:2parts. Acid residue is removed by two 10 minute washes with deionised water and the coverslip is dried under a flow of nitrogen (*N*_2_). A 50 μL solution of 10 nm NP-A-633 is drop-deposited onto the coverslip and after waiting two minutes, excess solution was removed by exposure to *N*_2_ flow. Samples are imaged under ICPM extended with fluorescence imaging capabilities to detect colocalised scattering from gold nanoparticles with fluorescent signatures. Overlap of the focal position of the two wavelengths used is ensured by passing the light through a single mode fibre prior to the beam splitter. Using the output from a single mode fibre as the source in combination with infinity corrected microscope objectives for out coupling and illumination respectively, ensures that chromatic shifts are eliminated and that fluorescence and scattering signals are colocalised to better than 100 nm. Figure 1(b) demonstrates the expected overlap of scattering and fluorescent signals using a reference sample of 10 nm fluorescent nanodiamond with Fig. 1(c,d) showing line scans 1 and 2 respectively. The scattering and fluorescent images are collected sequentially without adjustment to the microscope beyond excitation wavelength, and are depicted here as an overlapped false colour image where scattering is presented in green in arbitrary units (A.U.) and fluorescence is presented in red, in counts per pixel (cts/px). The characteristic four leaf clover pattern presented in the scattering signature results due to the detection of the scattered optical signal from gold nanoparticles under ICPM as discussed in earlier work^17^.

**Figure 2:**
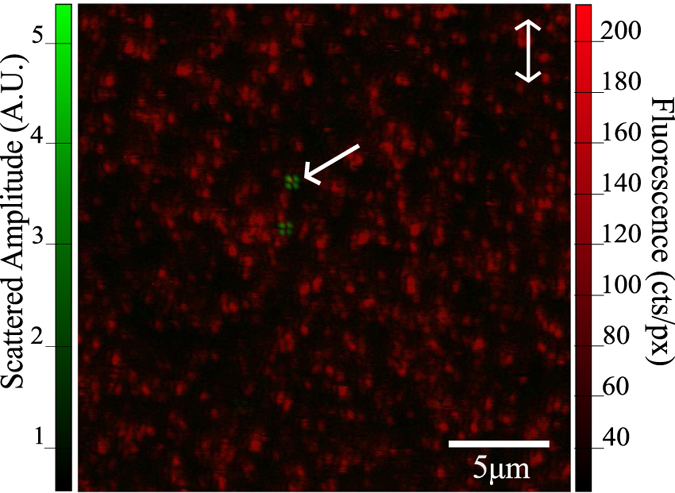
Scattering and fluorescent signal produced by unpurified NP-A-633 are overlayed and presented in a false colour image (green and red respectively). Images are captured at a pixel resolution of 45 nm/px × 49 nm/px over an imaging area of 23.2 μm × 25.0 μm at 512 pixels × 512 pixels collected at a scan rate of 0.4 s/line.

After deposition of the CLEM label following the procedure detailed above an area is scanned at a pixel resolution of ~47 nm/px with a fluorescence integration time of 0.55 ms. From the data presented in Fig. 2, we can see that even at low concentration of scattering nano-objects, deposited as received without purification steps, there exists a large population of unbound fluorophores to the extent that there is a fluorescent background in which we are unable to clearly resolve individual fluorescent spots; highlighting that this particular sample contains well over 100 fluorophores for every scattering particle. Interestingly, despite the high density of fluorescent signal, no signal is observed to colocalise with the scattering signatures. Prior to further investigation we endeavoured to reduce the background population of free fluorophores to enable imaging at a higher concentration of deposited scattering objects. A solution of 10 nm NP-A-633 underwent three cycles of centrifugation at 15,700 g for 20 minutes, between cycles the supernatant was removed and the solution re-dispersed in 200 μl of deionized water via a 20 minute sonication.

**Figure 3:**
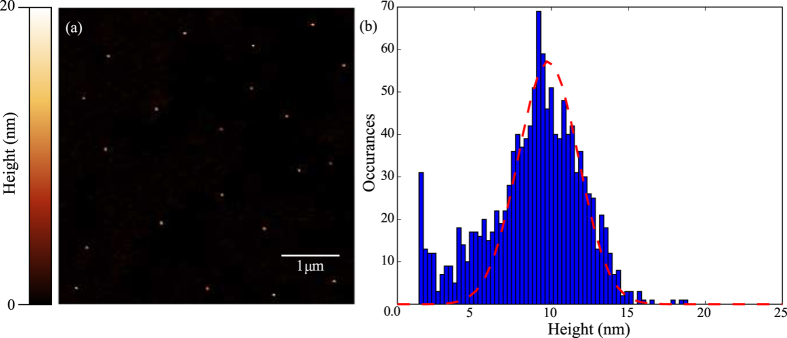
(**a**) Tapping-mode AFM image of purified 10 nm NP-A-633 after three cycles of centrifugation and re-dispersal at a pixel resolution of 9.76 nm/px × 9.76 nm/px. (**b**) A histogram of observed particles reveals a mean particle height of 9.80 nm with a FWHM (red dotted line) of 4.6 nm over a sample size of 1,258 particles.

As evidence that the purified 10 nm NP-A-633 remain isolated and spatially separated following the centrifugation and deposition procedure described above, a typical area imaged under tapping-mode atomic force microscopy (AFM) in air with a lateral pixel resolution of 9.76 nm/px is presented in Fig. 3(a) for an imaging size of 5.0 μm × 5.0 μm. By adjusting the dilution concentration, a desired inter-particle distance of greater than 500 nm is achieved to ensure that typically only a single particle is present in the focus for imaging under a diffraction limited approach. A survey of 1,258 particles over 14 randomly selected areas reveals a mean particle height of 9.8 nm with a FWHM of 4.6 nm in good agreement with the expected distribution as specified by the manufacturer, BBI Solutions, UK.

**Figure 4:**
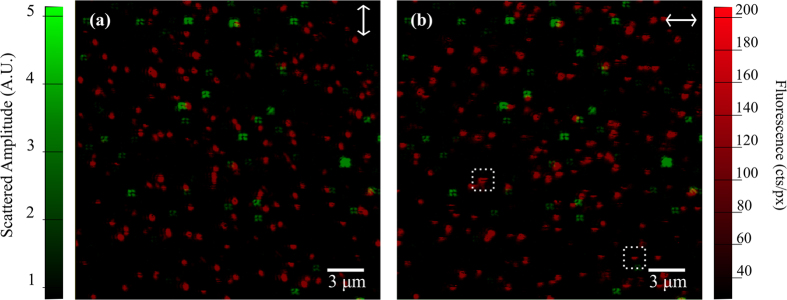
(**a**) False colour images of scattered (green) and fluorescent (red) signals are overlapped to facilitate observation of colocalisation events. (**b**) Results are also presented for the orthogonal illumination polarization to ensure all fluorescent molecules are excited. The dashed boxes identify blinking and bleaching events, evidence of single molecule detection. The applied optical power for *λ* = 532 nm is 14.9 μW and for *λ* = 632 nm the excitation power was measured as 7 μW. Images are captured at a pixel resolution of 45 nm/px × 49 nm/px over an imaging area of 23.2 μm × 25.0 μm at 512 pixels × 512 pixels collected at a scan rate of 0.4 s/line.

Results of imaging a sample of 10 nm NP-A-633 after achieving a sufficiently low level of unbound fluorophores are presented in Fig. 4(a). Observation of the scattered signal detected through ICPM shows the characteristic four lobe pattern associated with cross-polarized detection through high NA objectives^17^. Note that the signal has been clipped to facilitate highlighting all scattering signatures, which causes brighter signatures to appear to deviate from the cloverleaf structure. The fluorescent signatures present as diffraction limited spots elongated along the incident polarization direction consistent with linearly polarized illumination through high NA objectives, where the incident polarization direction is indicated by the arrow in the top right of the image; corresponding to x, y-polarization for Fig. 4(a,b). Figure 4(b) shows the same area where the fluorescence is captured under an illumination polarization rotated by 90^°^ to ensure all fluorescent molecules are excited and imaged regardless of the in-plane orientation of their absorption dipole. Due to rotation of our polarizing components, a small lateral shift in the detected image for orthogonal polarizations will occur when switching between incident polarization states. Images are aligned using the scattering image collected at *λ* = 532 nm for both illumination polarizations to correct for this effect.

Comparing subsequent fluorescent images, evidence of blinking (single dark pixels or dark lines through fluorescent signatures) and bleaching events (extinguishing of the fluorescent signature) are highlighted in the dashed boxes as evidence for detection levels down to single molecule sensitivity. Visual inspection of this figure shows no colocalisation between scattering and fluorescent signatures. To enable a full analysis, the fluorescent count rate originating from a single fluorescent emitter is needed independent of in-plane absorption dipole orientation. To do so, fluorescent signatures are identified for two orthogonal incident polarizations by applying a tracking algorithm to each fluorescent image. Positions of fluorescent signals are defined by local maxima in the fluorescent image above a threshold value of 20 cts/px and are collected for both incident polarization orientations. Pairs of fluorescent signatures are collected using the orthogonal polarization images by location within an arbitrarily chosen distance of approximately half the diffraction limit, 0.17 μm. In total 1,578 signature pairs are collected giving the fluorescent signal K under x and y polarizations respectively. An arbitrarily oriented dipole in free space defined in terms of Euler angles *φ* and *θ* with linear polarized excitation (*E*), produces a fluorescent signal (*K*) following:


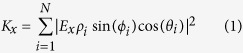



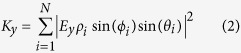


for x and y polarized light respectively, where *ρ*_*i*_ represents the dipole moment and, *φ*_*i*_ and *θ*_*i*_, the Euler angles of each observed fluorescent molecule. To retrieve *K*_*xy*_, the fluorescent emission that only depends on the out-of-plane angle *φ*, the peak fluorescence signatures from the two orthogonal polarization images is summed correcting for any difference in excitation powers.


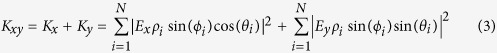



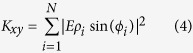


**Figure 5:**
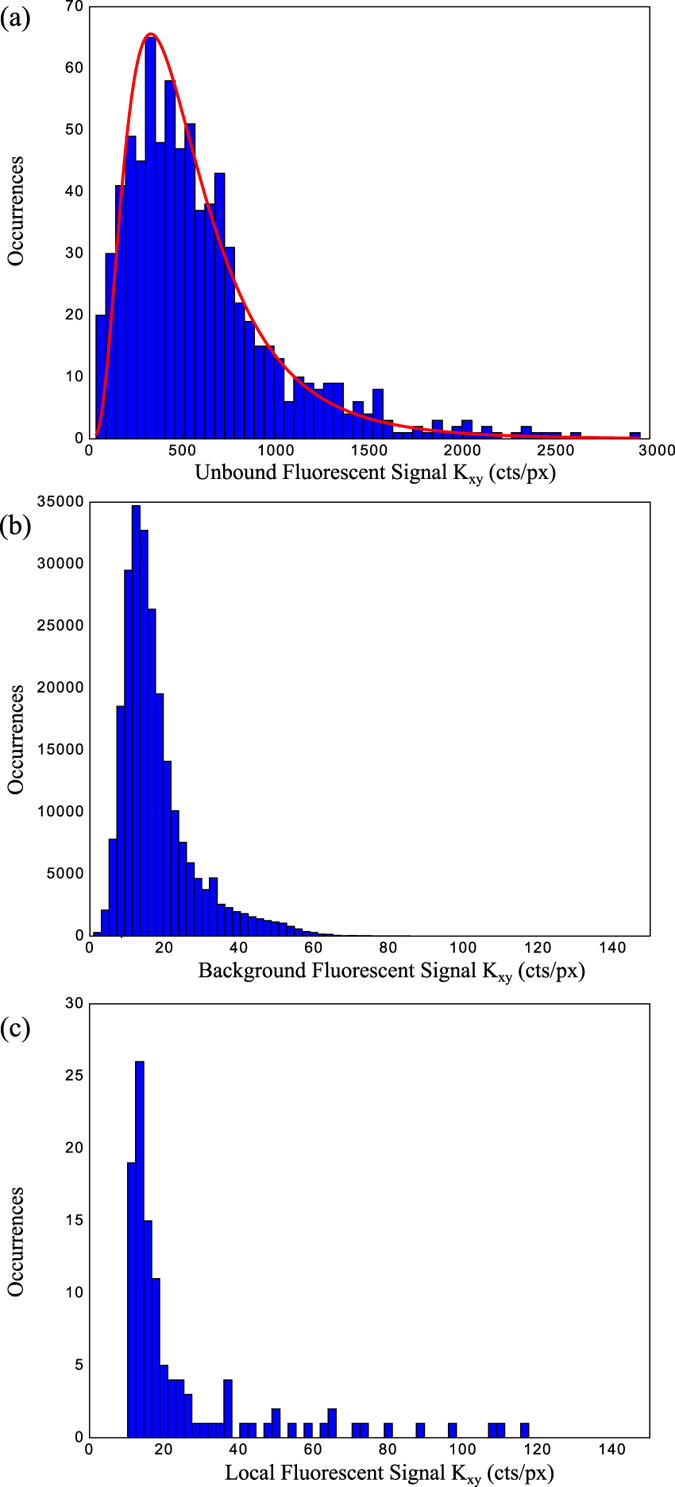
(**a**) A histogram of the fluorescent signals independent of in-plane dipole orientation (*K*_*xy*_) not associated with scattering signatures (unbound). (**b**) The distribution in fluorescent background counts further than 0.17 μm away from any identified scattering or fluorescent signatures. (**c**) The mean fluorescence localised within 0.17 μm of each scattering signature.

Applying the mathematical formalism above, equations (3,4), to all detected unbound fluorophores by excluding any fluorescent signals within 0.17 μm of a scattering signal, we find a peak in the histogram data of fluorescent count rate per pixel at 328 cts/px; Fig. 5(a). The count rate signal follows a log-normal distribution (red) and corresponds to the fluorescent signal liberated by a single fluorescent signature independent of in-plane dipole direction^18^. The fluorescent count rate of pixels not associated with fluorescent signatures (background counts) is presented as a histogram in Fig. 5(b) as a comparison to the fluorescent signal arising from the fluorophores, where corresponding fluorescent pixel areas have been used to compute the fluorescent signal independent of in-plane dipole orientation as above. Here the mean count rate is 17 cts/px with a trailing tail to higher values in a survey of 23,000 pixel pairs showing a signal to background of ~19 in these measurements.

In order to quantify the frequency of colocalisation events between scattering and fluorescent signals, a statistical survey is conducted for six randomly selected areas. As described in earlier work^12^, a machine-learning algorithm is employed to locate the centre of each scattering signature that matches well with the expected four leaf distribution due to detection under ICPM^17^. For the localisation of each detected scattering signature, the average fluorescent response within a 0.17 μm radius (approximately the radius of the diffraction limited spot at 532 nm) of each scattering centre is collected for both incident polarization images, which are combined following the mathematical treatment above to return the orientation-independent fluorescent signal associated scattering signatures; Fig. 5(c). This analysis enables the comparison of fluorescent levels associated with scattering signatures to the background fluorescent level. Over the observed 123 particles in these areas, we find a peak in the histogram at 17.5 cts/px, approximately the background pixel count rate, though proportionally higher rate of fluorescent signatures in the trailing tail compared to the histogram shown in (b). Close inspection of each of these higher values shows that they do not result from a fluorescent signature at the centre of the scatterer but result from a nearby fluorescent signature; positioned further away than 0.17 μm. Inspection of Fig. 5(a,b) hence shows that there is a complete absence of colocalisation between scattering and fluorescent signatures and that the level of *residual* localised fluorescence corresponding to scattering positions is approximately the background fluorescent rate. As we don’t see any colocalised signatures having a signal to background of ~19 this shows that the gold nanoparticle quenches the fluorescence signal by ~95% or less likely that the complex has dissociated. Furthermore, DOSY NMR analysis of a NP-A-633 sample shows Streptavidin-Alexa-633 complex with diffusion coefficients consistent with nanoparticle-bound systems with different degrees of substitution. These suggests that the absence of fluorescent colocalisation indeed arises due to quenching rather than disassociation between fluorescent marker and gold nanoparticle (see SI).

We believe that this paper shows that it is essential that any CLEM probe is properly examined with the necessary controls in order to check whether the probe behaves in the expected or predicted manner; not solely in terms of biological interaction of the probe but also in regards to photophysical behaviour and detection under multiple imaging modalities. We have previously shown how the former is essential for the use of Quantum dots^1^ where tagging Quantum dots with transferrin leads to miss-sorting. It may in some cases be necessary to only use fluorescence and generate a very high precision overlay^19^,^20^. Alternatively, there is the promising possibility of using single gold particles as the marker for both LM and EM, by leveraging ICPM or Four Wave Mixing^21^ as the light imaging modality or through detection of the luminescent blinking of gold nanoparticles^22^ potentially with reduced background by using the anti-Stokes emission^23^. Although we find here that gold-conjugated fluorophores are not suitable fiducial markers, there is evidence that the length of the linker may affect quenching efficiency with a larger spacer potentially even enhancing fluorescence emission^24^,^25^. Here we are unable to quantify this separation between fluorophore and gold nanoparticle in the used 10 nm NP-A-633 due to the commercial nature of the probe. A detailed study of the influence of the spacer and the suitability of such a larger probe for CLEM is the subject of further work.

## Conclusion

We have applied ICPM to the detection of a CLEM label (10 nm NP-A-633) to characterise the level of colocalisation between scattering signatures of gold nanoparticles and fluorescent signatures of Alexa-633. Characteristic signal levels are detected for 10 nm gold nanoparticles and fluorescent signatures are detected at single molecule sensitivity. However, we observe no colocalisation between scattering and fluorescent signatures hinting that metallic nanoparticle-single fluorophore systems should be treated cautiously when selected as probes for CLEM^1^. This result confirms earlier results^10^ in bulk solution that binding single fluorophores to metallic nanoparticles results in strong quenching of the fluorescence. Further work applying the technique presented herein is in progress to aid in the development of CLEM labels and that would allow for fast characterization at the level of the individual probe.

## Additional Information

**How to cite this article:** Miles, B. T. *et al*. Direct Evidence of Lack of Colocalisation of Fluorescently Labelled Gold Labels Used in Correlative Light Electron Microscopy. *Sci. Rep.* **7**, 44666; doi: 10.1038/srep44666 (2017).

**Publisher's note:** Springer Nature remains neutral with regard to jurisdictional claims in published maps and institutional affiliations.

## Supplementary Material

Supporting Information
