# Supplementary material for: Direct Evidence of Lack of Colocalisation of Fluorescently Labelled Gold Labels Used in Correlative Light Electron Microscopy
Source: Sci Rep. 2017 Mar 20;7:44666. doi: 10.1038/srep44666 (PMC5357795; doi:10.1038/srep44666)
Supplement: Supporting Information [file srep44666-s1.pdf]

Supporting Information for: Direct Evidence of Lack of Colocalisation of Fluorescently Labelled Gold Labels Used in Correlative Light Electron Microscopy

Benjamin T. Miles[1,+], Alexander B. Greenwood[1,+], David Benito-Alifonso[2], Hugh Tanner[3,5], M. Carmen Galan[2], Paul Verkade[4,5], and Henkjan Gersen[1,3,*]

[1]Nanophotonics and Nanophysics Group, H.H. Wills Physics Laboratory, University of Bristol, Bristol, BS8 1TL, UK
[2]School of Chemistry, University of Bristol, Cantock's Close, Bristol, BS8 1TS, UK
[3]Bristol Centre for Functional Nanomaterials, H.H. Wills Physics Laboratory, University of Bristol, Bristol, BS8 1TL, UK
[4]Wolfson Bioimaging Facility, University of Bristol, Bristol, BS8 1TD, UK
[5]School of Biochemistry, University of Bristol, Bristol, BS8 1TD, UK
[*]h.gersen@bristol.ac.uk

Diffusion ordered NMR spectroscopy (DOSY) measurements were performed using a Varian VNMRS500 spectrometer with a direct probe at 298 K. A pulsed gradient duration of 2 ms incremented from 3.9 to 45.5 G cm^{-1} in 15 steps and a diffusion time of 50 ms. DOSY measurements of a sample of NP-A-633 showed the presence of a set of signals corresponding to Streptavidin-Alexa633 with diffusion coefficients between $0.3\text{-}0.5\text{x}10^{-10}$ ms^{-2}. Peaks at higher diffusion coefficients corresponding to unbound citrate ($3.2\text{x}10^{-10}$ ms^{-2}), used to stabilize gold nanoparticles, were also observed.

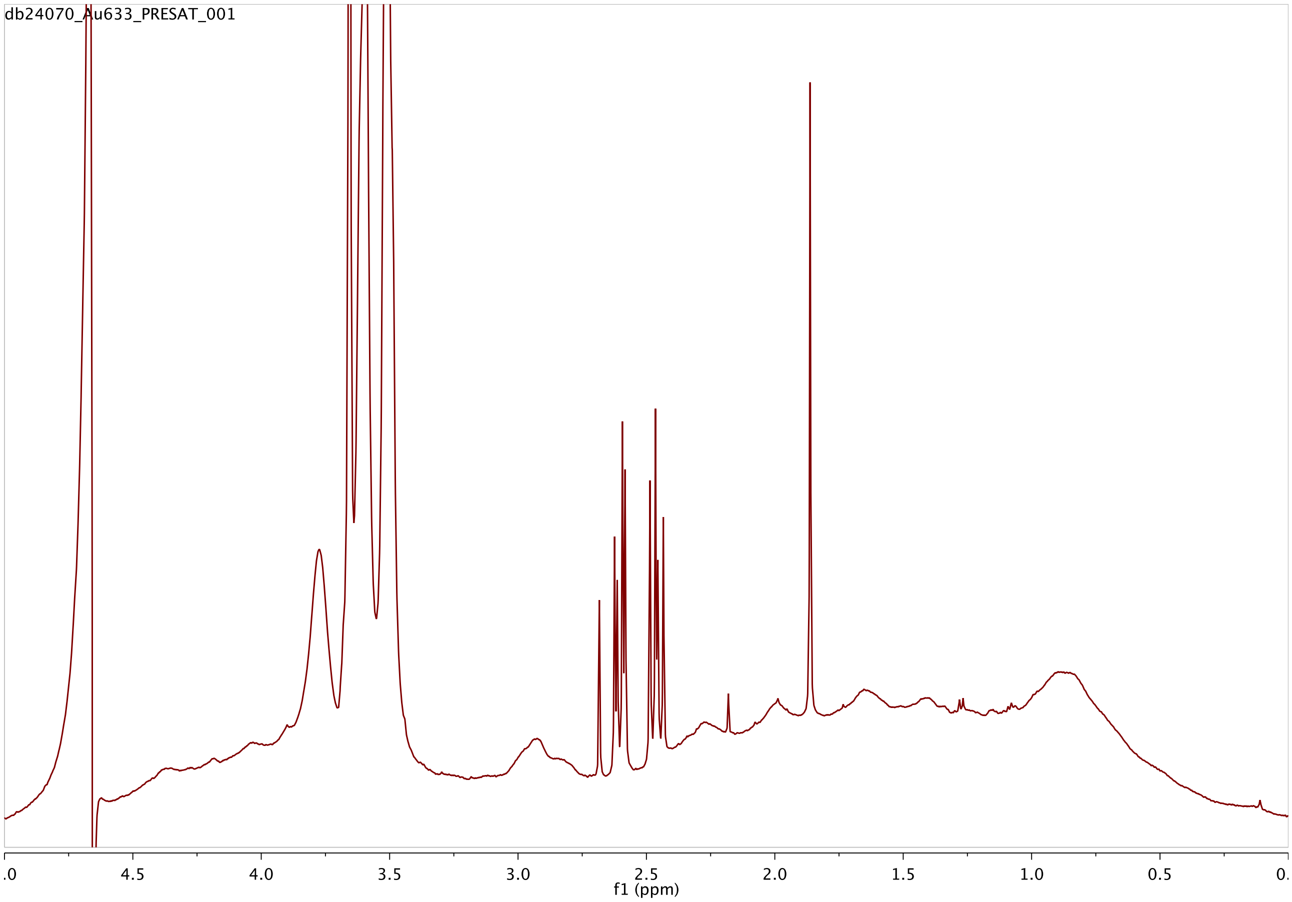

Figure 1. H NMR of Streptavidin-Alexa633

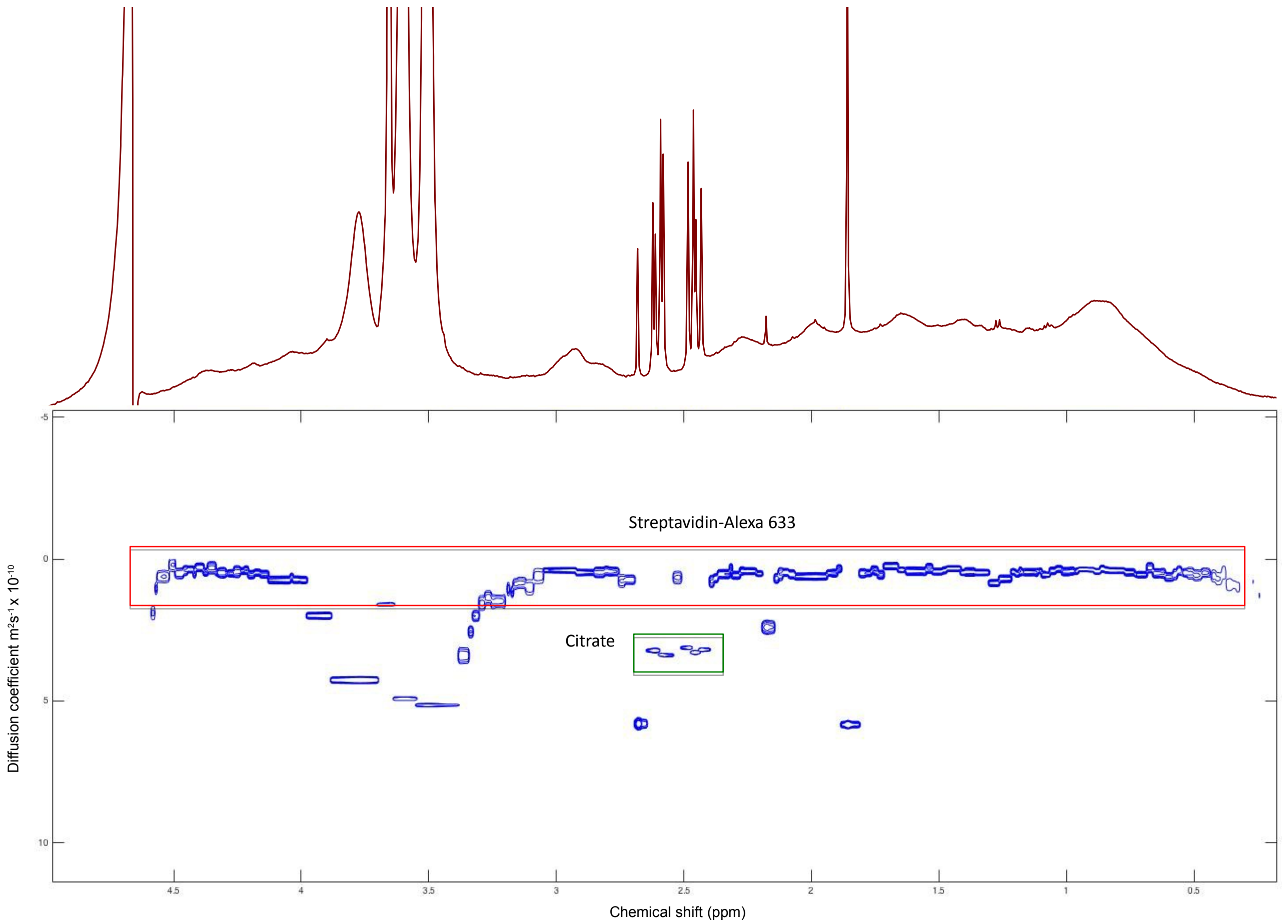

Figure 2. DOSY of NP-A-633
